# Cost Effectiveness of Cryptococcal Antigen Screening as a Strategy to Prevent HIV-Associated Cryptococcal Meningitis in South Africa

**DOI:** 10.1371/journal.pone.0069288

**Published:** 2013-07-19

**Authors:** Joseph N. Jarvis, Thomas S. Harrison, Stephen D. Lawn, Graeme Meintjes, Robin Wood, Susan Cleary

**Affiliations:** 1 Department of Clinical Research, Faculty of Infectious and Tropical Diseases, London School of Hygiene and Tropical Medicine, London, United Kingdom; 2 Desmond Tutu HIV Centre, Institute of Infectious Disease and Molecular Medicine, University of Cape Town, Cape Town, South Africa; 3 Research Centre for Infection and Immunity, Division of Clinical Sciences, St. George’s University of London, London, United Kingdom; 4 Department of Medicine, University of Cape Town, Cape Town, South Africa; 5 Clinical Infectious Diseases Research Initiative, Institute of Infectious Disease and Molecular Medicine, University of Cape Town, Cape Town, South Africa; 6 Department of Medicine, Imperial College London, London, United Kingdom; 7 Health Economics Unit, University of Cape Town, Cape Town, South Africa; Glaxo Smith Kline, Denmark

## Abstract

**Objectives:**

Cryptococcal meningitis (CM)-related mortality may be prevented by screening patients for sub-clinical cryptococcal antigenaemia (CRAG) at antiretroviral-therapy (ART) initiation and pre-emptively treating those testing positive. Prior to programmatic implementation in South Africa we performed a cost-effectiveness analysis of alternative preventive strategies for CM.

**Design:**

Cost-effectiveness analysis.

**Methods:**

Using South African data we modelled the cost-effectiveness of four strategies for patients with CD4 cell-counts <100 cells/µl starting ART 1) no screening or prophylaxis (standard of care), 2) universal primary fluconazole prophylaxis, 3) CRAG screening with fluconazole treatment if antigen-positive, 4) CRAG screening with lumbar puncture if antigen-positive and either amphotericin-B for those with CNS disease or fluconazole for those without. Analysis was limited to the first year of ART.

**Results:**

The least costly strategy was CRAG screening followed by high-dose fluconazole treatment of all CRAG-positive individuals. This strategy dominated the standard of care at CRAG prevalence ≥0.6%. Although CRAG screening followed by lumbar puncture in all antigen-positive individuals was the most effective strategy clinically, the incremental benefit of LPs and amphotericin therapy for those with CNS disease was small and additional costs were large (US$158 versus US$51per person year; incremental cost effectiveness ratio(ICER) US$889,267 per life year gained). Both CRAG screening strategies are less costly and more clinically effective than current practice. Primary prophylaxis is more effective than current practice, but relatively cost-ineffective (ICER US$20,495).

**Conclusions:**

CRAG screening would be a cost-effective strategy to prevent CM-related mortality among patients initiating ART in South Africa. These findings provide further justification for programmatic implementation of CRAG screening.

## Introduction

Cryptococcal meningitis (CM) is one of the leading causes of death in HIV-infected patients in Africa. CM accounts for between 33% and 63% of all adult meningitis in southern Africa [1]–[3], and acute mortality ranges from 24% to 50% [4]–[9]. As a result CM is estimated to cause in excess of 500,000 deaths annually in sub-Saharan Africa [10]. Prevention strategies are therefore of great public health importance. Recent data from South Africa suggest that the vast majority of patients who develop CM are already in care with an established HIV diagnosis [11] and that a sizeable proportion present following initiation of antiretroviral therapy (ART) [4], [6]. Thus, opportunities exist for preventive interventions.

Timely initiation of (ART) resulting in immune reconstitution is clearly the most effective strategy for preventing all HIV-related opportunistic infections [12], and a marked decline in the incidence of cryptococcal disease was seen in the developed world following the introduction of effective ART [13]–[15]. Unfortunately, despite recent progress in expanding access to ART in South Africa, a substantial proportion of patients still present late with advanced immunodeficiency and high risk of new AIDS events and mortality [16]. Thus, preventive interventions implemented immediately before or concomitantly with ART, could be an effective initial strategy in the treatment of patients with advanced HIV, allowing patients the best chance at long-term disease free survival.

Universal fluconazole primary prophylaxis in areas of high incidence of cryptococcal disease has been shown to be highly effective at reducing the incidence of CM [17]–[23]. However, no study has yet shown a significant reduction in mortality [23]. The inefficiency of this strategy with regard to the large numbers of patients requiring treatment [17], [24], high cost [25]–[29], and concerns regarding drug resistance [29]–[32], has meant that such a strategy has never been widely implemented.

More recently it has been shown that the vast majority of patients at risk of developing CM during ART can be identified at the time of entry into ART services by screening for sub-clinical infection using simple and low-cost cryptococcal antigen (CRAG) immunoassays on blood samples [33]. Current CRAG immunoassays are highly sensitive and specific, and CRAG screening at ART initiation has been shown to be 100% sensitive and 96% specific for predicting development of CM during the first year of ART [33]. Patients identified during CRAG screening pre-ART could be targeted with “pre-emptive” therapy to prevent the development of severe disease. This strategy enables identification of a limited number of patients at risk who can then receive intensive investigation and treatment, avoiding the costs of widespread and unnecessary drug exposure and the associated risk of development of drug resistance [32].

To inform policy makers considering programmatic implementation of CM prevention strategies we performed a cost-effectiveness analysis of four different strategies to prevent cryptococcal meningitis in individuals initiating ART in South Africa with CD4 cell-counts <100 cells/µl: ART alone, with no screening or prophylaxis (the current standard of care); universal primary fluconazole prophylaxis; CRAG screening with targeted high-dose fluconazole treatment for all patients testing positive; or CRAG screening with subsequent lumbar puncture (LP) for those testing positive and treatment either using amphotericin B for those with infection of the central nervous system (CNS) or high-dose fluconazole for those without.

## Methods

### Study Design

Using primarily South African data on CRAG prevalence, CM incidence in ART programmes, CM-related mortality and health service costs we modeled the cost-effectiveness of four strategies in patients with CD4 cell-counts <100 cells/µl: 1) no screening or prophylaxis (standard of care), 2) universal primary prophylaxis with fluconazole 200 mg daily, 3) CRAG screening with high-dose fluconazole treatment for all patients testing positive (800 mg fluconazole daily for two weeks, followed by 400 mg daily for eight weeks, then 200 mg daily maintenance) and 4) CRAG screening with lumbar puncture for all patients testing positive, amphotericin-B 1 mg/kg/day for two weeks for those with CNS disease and fluconazole 800 mg daily for two weeks for those without, in both cases followed by fluconazole 400 mg daily for eight weeks, then 200 mg daily maintenance as above. A CD4 count cut-off of 100 cells/µl was used as nearly all cases of CM developing following ART initiation occur in this patient group [33], and prevention strategies will be targeted at this population. Incremental costs were assessed from the provider’s perspective. We only included the health care costs associated with the treatment and prevention of CM. Outcomes were defined as life-years gained. Given our restricted scope of provider costs, we also only considered deaths from CM within our estimates of life-years gained. The main summary measures used for presenting results were annual costs, annual life years and incremental cost effectiveness ratios (ICER). The latter is a ratio that summarizes the difference in costs to the difference in life years. However, when a more effective intervention is less costly than a comparator, it is said to dominate the comparator, and no ICER is calculated.

### Model

**Figure 1:**
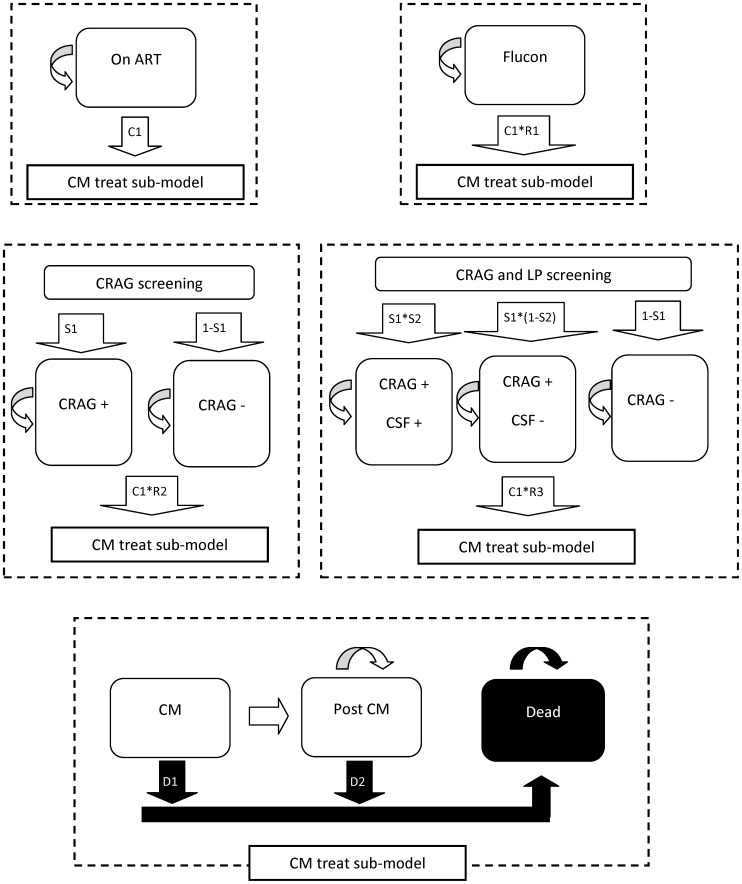
Simplified markov model structure. Transition probabilities are listed as C1, C2, S1 etc. Variable names, descriptions and values are derived from table 1, as follows: ***S1*** - Proportion with subclinical CM at baseline, CD4<50 cells/µL = 0.13, CD4 50–100 cells/µL = 0.03. ***S2*** - Of those with subclinical CM, Proportion with CSF infection = 0.5. ***P1*** - Proportion starting ART with CD4<50 cells/µl = 0.5. ***C1*** - Probability of developing CM, by baseline CD4 category and time on ART: *CD4<50 cells/µl = *252 per 1000 patient years (pyo) up to 3 months on ART, 72 per 1000 pyo 4–6 months on ART, 36 per 1000 pyo 7–9 months on ART, 0 per 1000 pyo 10–12 months. *CD4 50–100 cells/µl* = 56 per 1000 pyo up to 3 months on ART, 16 per 1000 pyo 4–6 months on ART, 8 per 1000 pyo 7–9 months on ART, 0 per 1000 pyo 10–12 months. ***R1*** - Relative risk of CM with low dose fluconazole prophylaxis = 0.21. ***R2*** - Relative risk of CM for CRAG positive taking high dose fluconazole prophylaxis = 0.1. ***R3*** - Relative risk of CM for CRAG positive with amphotericin for CSF positive patients, fluconazole for CSF negative patients = 0. ***D1*** - Probability of dying of acute CM = 45% dead at 1 month with CM. ***D2*** - Probability of dying of CM within 1 year = 55% dead at 12 months on ART.

We modeled the costs and outcomes of our alternative strategies within a Markov model using a monthly cycle length, and ran the model for 12 cycles in order to calculate annual costs and annual life years. While we have not used our model to extrapolate data beyond the period of observed follow-up, a Markov modeling framework has been chosen as it allows for the synthesis of data from secondary sources and for sophisticated sensitivity analyses to be performed [34]–[35]. A simplified version of the structure of the Markov model is presented in Figure 1. (The full model is presented in figure S1).

### Transition Probabilities (see Table 1)

The CD4 cell count distribution of patients entering ART programmes (split into ≤50 cells/µL and 51–100 cells/µL), was from a cohort of patients entering the ART service based at a public sector community clinic in Cape Town [33]. The probability of a positive serum CRAG at baseline was determined using data from seven African and two international cohort studies [33], [36]–[42] (and Govender unpublished), and the probability of CRAG positive patients having evidence of CNS disease at LP derived from the four published cohorts [36]–[37], [41]–[42]. The probability of developing CM was derived from two separate lines of data. Initially data regarding the clinical course of untreated cryptococcal antigenaemia in patients initiating ART was used to ascertain the proportion of CRAG positive patients, stratified by CD4 cell count, who subsequently develop CM [33], [39]. These probabilites were applied to the CRAG prevalence figures described above and were then verified using reported cryptococcal incidence data from sub-Saharan Africa [33], [43]–[45]. In addition to stratification into two categories of CD4 cell count to enable sensitivity analysis (≤50 cells/µL and 51–100 cells/µL), transition probabilities relating to the development of CM were varied according to time on ART to account for CD4 cell count increases, and the acompanying reduction in risk of developing CM. This time stratification was into 3 month blocks according to time from ART initiation, and the data used was from a large South African cohort [45]. The probability of death in patients developing CM was derived from six South African studies [4]–[6], [8]–[9], [46] and data from the South African Mycology Reference Unit, National Institute for Communicable Diseases (Govender, unpublished), with a slight weighting towards those reflecting programmatic outcomes rather than research conditions.

**Table 1:** Baseline input assumptions and transition probabilities.

Input	Value	*Reference*
Mean CD4 count	**50 cells/µl**	*Jarvis et al. CID * ***2009*** * 48:856-62; Longley * ***2011*** * unpublished*
Prevalence of cryptococcal antigenaemia	**8%**	*Desmet et al. AIDS * ***1988*** * 3:77-8; Tassie et al. JAIDS * ***2003*** * 33:411-2; Liechty et al. TMIH * ***2007*** * 12:929-35; Micol et al. JAIDS * ***2007*** * 45:555-9; Jarvis et al. CID * ***2009*** * 48:856-62; Pongsai et al. J Infect * ***2010*** * 60:474-7; Meya et al. CID * ***2010*** * 51:448-55; Govender * ***2011*** * unpublished*
Incidence of CM*	**55/1000 pyo**	*French et al. AIDS * ***2002*** * 16:1031-8; Holmes et al. JAIDS * ***2006*** * 42:464-9; Jarvis et al. CID * ***2009*** * 48:856-62; Jarvis et al. CID * ***2010*** * 51:1463-5*
*CD4<50 cells/µl*		
0–3 months on ART	**252/1000 pyo**	*Jarvis et al. CID * ***2009*** * 48:856-62; Jarvis et al. CID * ***2010*** * 51:1463-5*
4–6 months on ART	**72/1000 pyo**	
7–9 months on ART	**36/1000 pyo**	
>10 months on ART	**0/1000 pyo**	
*CD4 50–100 cells/µl*		
0–3 months on ART	**56/1000 pyo**	*Jarvis et al. CID * ***2009*** * 48:856-62; Jarvis et al. CID * ***2010*** * 51:1463-5*
4–6 months on ART	**16/1000 pyo**	
7–9 months on ART	**8/1000 pyo**	
>10 months on ART	**0/1000 pyo**	
Duration of hospitalization with CM	**15 days (13–20)**	*Jarvis et al. J Infect * ***2010*** * 60:496-498*
Mortality of CM	**45% acute; 55% at 1 year**	*Jarvis et al. J Infect * ***2010*** * 60:496-498; Bicanic et al. CID * ***2007*** * 45:76-80; Bicanic et al. CID * ***2008*** * 47:123-30; Jarvis et al. CROI * ***2011*** * P-123; Lightowler et al. PLoS ONE * ***2010*** * 5:e8630; Lessells et al. SAMJ * ***2011*** * 101:251-252; Govender * ***2011*** * unpublished*
Proportion CRAG +ve with CNS disease	**50%**	*Desmet et al. AIDS * ***1988*** * 3:77-8; Tassie et al. JAIDS * ***2003*** * 33:411-2; Pongsai et al. J Infect * ***2010*** * 60:474-7; Micol et al. JAIDS * ***2007*** * 45:555-9*
Relative risk of CM with primary fluconazole prophylaxis	**0.21**	*Chang et al. Cochrane Database Syst Rev * ***2005*** * CD004773.*
Relative risk of CM with CRAG screening and highdose fluconazole	**0.1**	*Meya et al. CID * ***2010*** * 51:448-55; Pongsai et al. J Infect * ***2010*** * 60:474-7; Micol et al. JAIDS * ***2007*** * 45:555-9; Feldmesser et al. CID * ***1996*** * 23:827-30; Yuen et al. CID * ***1994*** * 19:579; Longley et al CID* **2008** 47:1556-61
Relative risk of CM with CRAG screening plus LP	**0.0**	

* The incidence of CM varied according to CD4 count strata, and time on ART to account for CD4 cell count increases and the acompanying reduction in risk of developing CM. The time stratification was into 3 month blocks according to time from ART initiation, and the data used to derive these probabilities was from a large South African cohort [45].

Data from a Cochrane meta-analysis were used to determine the relative risk of developing CM with primary fluconazole prophylaxis. CRAG screening immediately prior to ART initiation was found to be 100% sensitive at identifying patients at risk of developing CM in our previously reported study [33]. Combining the data from all available published global literature we assumed a relative risk of 0.1 for the development of CM in CRAG-positive patients receiving high dose fluconazole pre-emptive treatment [24], [39], [41]–[42], [47]. The most comprehensive management approach was assumed to be 100% effective at preventing subsequent symptomatic CM. This entailed doing LPs on all patients testing CRAG-positiveand treatment either with amphotericin for those with evidence of CNS disease or high-dose fluconazole for those without.

### Costs (see Table 2)

A micro costing approach was used: the ingredients of each intervention (e.g. number of lab tests, dosages of medication etc) were combined with the unit cost of each ingredient to calculate a monthly cost per Markov state, and ultimately a cost per patient-year within each intervention arm. Costs included prophylactic medication (fluconazole, at varying doses), screening costs (CRAG screening test plus titre, lumbar punctures), and treatment costs for those developing CM (including inpatient care, outpatient department visits, inpatient medication and tests). Medication costs were from government tender prices, test costs were from the National Health Laboratory Services, and lumbar puncture costs were based on the Uniform Patient Fee Schedule. The overhead and staff cost per inpatient day at the secondary level and the overhead and staff cost per outpatient department visit was taken from Cleary et al [48]. The overhead components of these costs were inflated using the Consumer Price Index, while clinical staff costs were recalculated using 2010 government salary scales. Costs were expressed in 2010 prices, and were converted to United States Dollars (US$) based on the average exchange rate between 1 January and 31 December 2010 (US$1 = ZAR7.34; www.oanda.com).

**Table 2:** Costs.

Markov state	Description	Resource usage	Unit cost (US$)*
On ART	General state for patients on ART	None	0
Flucon	Tunnel states to capture time-variant costs of primary fluconazole prophylaxis	Fluconazole 200 mg per day for 12 months	497.50
CRAG screening	Temporary (1 cycle) state to capturecosts of CRAG screening	1 cryptococcal antigen test plus titre	10.86
CRAG +	Tunnel states to capture time-variantcosts of targeted fluconazole for CRAGpositive patients	Fluconazole 800–1200 mg per day for 14 days; Fluconazole 400 mg per day for56 days; Fluconazole 200 mg per day for remaining period up to 12 months	631.07
CRAG −	General state for CRAG negativepatients	None	0
CRAG and LPscreening	Temporary (1 cycle) state to capture costs of CRAG and LP screening	1 cryptococcal antigen test plus titre	10.86
		For those positive: 1 lumbar puncture	17.04
CRAG +CSF +	Tunnel states to capture time-variant costs of inpatient and fluconazole treatment for CRAG and CSF positive patients	Hotel costs over 15 inpatient days	2,266.03
		4 full blood counts	26.59
		1 litre saline IV over 14 inpatient days	19.04
		50 mg amphotericin B over 14 inpatient days	54.32
		1 lumbar puncture	17.04
		4 creatinine, electrolyte, urea tests	53.02
		Fluconazole 400 mg per day for 56 days; Fluconazole 200 mg per dayfor remaining period up to 12 months	554.74
CRAG +CSF −	Tunnel states to capture time-variant costs of targeted fluconazole for CRAG positive and CSF negative patients	Fluconazole 800–1200 mg per day for 14 days; Fluconazole 400 mg per day for56 days; Fluconazole 200 mg per day for remaining period up to 12 months	631.07
CRAG −	As above		
CM	Temporary (1 cycle) state for patients with CM	Hotel costs over 15 inpatient days	2,266.03
		4 full blood counts	26.59
		1 litre saline IV over 14 inpatient days	19.04
		50 mg amphotericin B over 14 inpatient days	54.32
		2 lumbar punctures	34.08
		4 creatinine, electrolyte, urea tests	53.02
		2 outpatient department visits	51.74
Post CM	Tunnel state for patients receiving secondary fluconazole prophylaxis after inpatient care for CM	Fluconazole 400 mg per day for 56 days; Fluconazole 200 mg per dayfor remaining period up to 12 months	554.74

* Medication costs were from government tender prices, test costs were from the National Health Laboratory Services, and lumbar puncture costs were based on the Uniform Patient Fee Schedule. The overhead and staff cost per inpatient day at the secondary level and the overhead and staff cost per outpatient department visit was taken from Cleary et al [48]. The overhead components of these costs were inflated using the Consumer Price Index, while clinical staff costs were recalculated using 2010 government salary scales. Costs were expressed in 2010 prices, and were converted to United States Dollars (US$) based on the average exchange rate between 1 January and 31 December 2010 (US$1 = ZAR7.34; www.oanda.com).

### Uncertainty and Sensitivity Analysis

The literature identifies two sources of uncertainty that are relevant to this study: the dataset, and the generalizability of results [49]. Uncertainty relating to the data has been assessed using probabilistic sensitivity analysis (PSA). Where possible, distributions (uncertainty intervals) were specified on utilisation, and transition probability variables, and uncertainty was captured by running 100,000 second-order Monte Carlo simulations. We have not assessed uncertainty associated with discounting as the time horizon of this study precluded any need for discounting. Finally, we have sought to strengthen the generalizability of our results through additional one- and multi-way simple sensitivity analyses. All analyses were run in TreeAge Pro 2006.

## Results

### Outcomes

Outcomes at the end of the twelve month cycle were determined using the transition probabilities listed in table 1. Risk of developing CM varied by baseline CD4 category and time on ART, with an overall probability of developing CM of 0.09 in patients with CD4 counts <50 cells/µL and 0.02 in those with CD4 counts of 50–100 cells/µL. Using these input parameters, in the standard of care strategy the probability of dying from CM during the first year of ART was 1.87% (95% CI 1.0–2.8%). This is in keeping with data suggesting that mortality during the first year of ART is in the range of 8–26% in resource limited settings, and that CM accounts for up to 20% of this mortality [16]. The most effective strategy for preventing mortality due to CM in this model was CRAG screening with lumbar puncture of all CRAG positive patients, followed by CRAG screening with high dose fluconazole, then primary prophylaxis (see table 3).

**Table 3:** Cost-effectiveness.

*Table 3* * (A).* Cost-effectiveness of strategies to prevent cryptococcal meningitis in South Africa								
	Mean cost (US$)		95% CI*	Life years		95% CI*		Incremental cost-effectiveness ratio (ICER)
CRAG screening with high dose fluconazole	**51.41**		39.14–64.46	0.9999		0.9992–1		––
CRAG screening with lumbar puncture	**157.53**		111.92–207.94	1				889,266.69
Standard of care	**207.36**		158.38–261.36	0.9813		0.9718–0.99		(Dominated) †
Universal primary prophylaxis	**582.41**		578.49–590.39	0.9996		0.9983–1		(Dominated)†
***Table 3*** *** (B).*** ** Sensitivity analysis: impact of varying the prevalence of cryptococcal antigenaemia in the population**								
		**Mean cost (US$)**			**Life years**		**ICER**	
**Decreasing baseline antigen prevalence to 6%**								
CRAG screening with fluconazole		**41.27**			0.999			
CRAG screening with lumbar puncture		**120.86**			1		889,185.26	
Standard of care		**157.07**			0.9859		(Dominated)	
Universal primary prophylaxis		**581.43**			0.9997		(Dominated)	
**Decreasing baseline antigen prevalence to 4%**								
CRAG screening with fluconazole		**31.14**			0.9999			
CRAG screening with lumbar puncture		**84.19**			1		889,087.45	
Standard of care		**105.75**			0.9905		(Dominated)	
Universal primary prophylaxis		**580.45**			0.9998		(Dominated)	
**Decreasing baseline antigen prevalence to 2%**								
CRAG screening with fluconazole		**21.00**			1			
CRAG screening with lumbar puncture		**47.53**			1		889,083.68	
Standard of care		**53.40**			0.9952		(Dominated)	
Universal primary prophylaxis		**579.47**			0.9999		(Dominated)	

Mean cost = Mean per-patient cost for prevention and/or treatment of CM, US$, during first year of ART.

Life years = Mean life expectancy one year after ART programme entry.

ICER = ratio of difference in cost to difference in outcome.

* Uncertainty interval.

† Higher cost than more effective option(s).

### Costs

**Figure 2:**
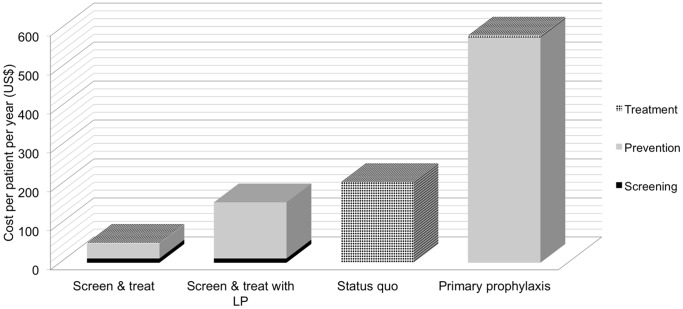
Cost Breakdown. The cost of each of the four strategies divided into screening costs, preventive treatment (or “prevention”) costs and treatment costs. Screening costs (black shading) include all costs associated with CRAG screening including the CRAG assay. Prevention costs (grey shading) include all costs associated with prevention including universal fluconazole in the primary prophylaxis strategy; and fluconazole pre-emptive treatment for CRAG positive patients, LPs, clinic visits and in-patient amphotericin for screened patients with CNS involvement in the screen and treat strategies. Treatment costs (cross-hatched shading) include all costs associated with treatment of CM in patients who develop clinical CM. Costs are as outlined in table 2, and expressed as mean cost per patient/year in the ART programme.

The costs associated with each Markov state are outlined in Table 2. The table indicates the resources that are needed for each of the screening, prophylactic and treatment options, as well as the full cost for each. Overall costs for each strategy, expressed as mean costs per patient/year in the ART programme, were US$51.41 for CRAG screening with targeted treatment of CRAG positive individuals with high dose fluconazole; US$157.53 for CRAG screening with lumbar puncture (LP) in all CRAG positive individuals and amphotericin B for those with evidence of central nervous system (CNS) infection, and high dose fluconazole for those without; US$207.36 for standard of care (no prevention intervention); and US$582.41 for universal primary prophylaxis (table 3). The cost of the standard of care strategy was driven almost entirely by inpatient treatment of patients developing CM, while in both the CRAG screening with targeted treatment of CRAG positive individuals with high dose fluconazole and primary prophylaxis strategies costs were almost exclusively for prevention in out-patient settings (figure 2).

### Cost-effectiveness

Over the twelve month cycle modelled, both screen and treat strategies were more effective and less expensive than the current standard of care and universal primary prophylaxis (i.e “dominated” – see table 3). CRAG screening with lumbar puncture in all CRAG positive individuals was the most effective strategy, and less costly than the current standard of care. However CRAG screening with targeted treatment of CRAG positive individuals with high dose fluconazole was only marginally less effective, while being considerably less expensive (US$ 51.41 versus US$157.53), leading to a high incremental cost-effectiveness ratio. Primary prophylaxis was more expensive and more effective than the current standard of care (US$582.41 per patient/year versus US$207.36, with an ICER of US$20,494.54 per life year gained).

### Sensitivity Analysis

**Figure 3:**
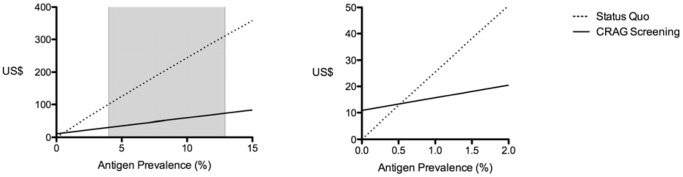
Sensitivity analysis by background antigen prevalence. The results of one-way sensitivity analysis varying the background cryptococcal antigen prevalence in patients entering ART programmes with CD4 cell counts <100 cells/µL. The cost of current standard of care (no prevention, or status quo) is shown by the dotted line, and the cost of the CRAG screening with targeted treatment of CRAG positive individuals with high dose fluconazole (no LPs) is shown by the solid line. The screen and treat strategy dominated the standard of care at antigen prevalences of 0.6% and higher. The shaded area represents the range of baseline CRAG prevalence figures reported in patients with CD4 counts <100 cell/µL at ART programme entry. Costs are expressed as mean cost per patient/year in the ART programme.

One-way sensitivity analyses were conducted to determine how sensitive the results were to changes in baseline CRAG prevalence (table 3 and figure 3) and rates of loss to follow-up in the ART programme. Increasing loss to follow-up rates to 50% increased the cost of CRAG screening with targeted treatment of all CRAG positive individuals with high dose fluconazole to US$135.07 and increased the probability of death due to CM to 0.8%; however the strategy still dominated the standard of care (the costs for which would remain basically unchanged). Assuming the baseline prevalence of cryptococcal antigenaemia was less than 1% had limited effect on the overall results, with CRAG screening with targeted treatment of all CRAG positive individuals with high dose fluconazole dominating the standard of care to CRAG prevalence of 0.6%, and CRAG screening with lumbar puncture dominating standard of care to CRAG prevalence of 2.5% (figure 3).

## Discussion

This study suggests that CRAG screening of blood from patients entering ART programmes and targeted treatment of those testing positive would be a cost-effective intervention in South Africa, saving both money and lives when compared to the current standard of care. Although the most effective strategy for preventing CM is CRAG screening followed by LP for all patients identified as having asymptomatic cryptococcal antigenaemia, the incremental benefit of performing LPs and giving amphotericin based therapy for those identified as having CNS disease is small, while the additional costs are large. Both CRAG screening strategies are more cost-effective than current standard of care. However the most feasible and economically most favourable strategy was to screen all patients entering ART programmes with CD4 cell counts less than 100 cells/µl for CRAG, with targeted treatment of all CRAG positive individuals with high dose fluconazole. From a public health perspective this may be the best and most feasible approach to implement, and sensitivity analysis demonstrates such a screen and treat strategy is both less costly and more effective than the current standard of care in settings with antigen prevalences as low as 0.6% - well below the lower reported antigen prevalence figures of 4% (figure 3). Given the small but important benefit gained at the level of the individual patient by performing LPs and offering more aggressive treatment, in well resourced settings where this is possible, it may also be an attractive strategy. Again this is less costly and more effective than the current standard of care with an antigen prevalence as low as 2.5%.

Universal primary fluconazole prophylaxis has previously been clearly shown to reduce the incidence of CM [22]–[23]. However, using South African costing and incidence data, this is the least cost-effective cryptococcal prevention strategy, not only costing considerably more than either CRAG screening approach, but also was less effective at preventing CM. The high cost of universal primary prophylaxis is primarily driven by the cost of preventive fluconazole, even though the lowest available generic drug prices are utilized in the analysis. This finding is in keeping with the results from prior cost-effectiveness analyses of fluconazole primary prophylaxis which have generally been unfavourable. Estimates range from US$70,000 to US$240,000 per quality adjusted life year (QALY) gained [25]–[28], even assuming high incidence rates and high efficacy of prophylaxis.

Two other published analyses have examined the cost effectiveness of CRAG screening and targeted preventive treatment, and in keeping with this analysis both concluded that antigen screening is a cost-effective strategy to prevent HIV-associated CM in patients with CD4 cell counts below 100 cells/µL [39], [50]. Meya et al estimated that in Uganda the number of patients who would need to be CRAG screened and fluconazole treated to prevent one case of CM was 11.3 at a cost of US$190, or, to save one life, 15.9 patients at a cost of US$266. This equated to US$21 per disability adjusted life year (DALY) gained [39]. An important caveat to these data is that no allowance was made for the avoided costs of in-patient amphotericin B treatment inherent in the screening strategy, hence the true cost-effectiveness of CRAG screening may have been underestimated [45]. Micol et al, in Cambodia, also found CRAG screening to be a cost-effective intervention, costing US$180 per life year gained when compared to no intervention. As in our South African analysis, primary fluconazole prophylaxis was shown to be less cost-effective than CRAG screening in Cambodia [50].

Our analysis differs from the two previous cost-effectiveness analyses in several important respects. We have modelled the impact of high dose fluconazole pre-emptive therapy in the CRAG screening arm, which is an approach based on the best evidence available and the strategy being proposed for implementation at a national level in South Africa (Southern African HIV Clinicians Society Guidelines, in preparation). We have also examined the impact on costs and outcomes of performing LPs as part of the CRAG screening intervention. These are strengths of the analysis as they address the practically relevant clinical and programmatic questions facing clinicians and policy makers. Further strengths of this analysis are the robustness of the underlying clinical and costing assumptions. Data on CRAG prevalence and outcomes are derived from the largest reported prospective cohort [33] and extensive southern African data on incidence, natural history and outcomes of cryprococcal infections. This has been combined with accurate costing data from the South African public health care sector [48], which while potentially limiting the generalizability of these data to settings outside southern Africa, provides the first detailed information for policy makers in the area of the world with the highest burden of cryptococcal disease.

As with all analyses of this type, the primary limitations relate to the necessity to make underlying assumptions based on data from a variety of sources. However the data from which the transition probabilities were generated is, wherever possible, from large and well-conducted clinical trials and cohort studies. Estimates of CRAG prevalence, cryptococcal incidence and cryptococcal disease outcomes were derived from a large body of published work. Outcomes without preventive measures in place reflect data reported from South Africa and the impact of primary prophylaxis was derived from a meta-analysis. Fewer data regarding the efficacy of CRAG screening were available; hence the estimates used are subject to greater uncertainty. However, the estimates used take into account all currently available evidence. The sensitivity analyses performed demonstrate that even with wide variations in CRAG prevalence, access to effective treatments and rates of follow-up, the key findings about relative costs and effectiveness remain unchanged.

A growing body of evidence is emerging to support the use of CRAG screening as a tool to prevent CM. This study adds further weight to this evidence, showing that CRAG screening would be a highly cost-effective intervention in South Africa, the country with the highest burden of HIV in the world. These findings, along with other reported studies [33], [45] strongly support the implementation of CRAG screening within the South African ART programme. The most cost-effective strategy of CRAG screening with targeted high dose fluconazole for those testing positive would be cost saving compared to the current standard of care, is practical, and easily implementable. Recently developed low-cost and simple point-of-care CRAG tests [51] have not been considered in this analysis as they were not available during the study period, but cost approximately two-fold less than the CRAG pricing used in this study. This promises to make CRAG screening feasible in an even wider range of settings and at lower cost. A further impact of implementing CRAG screening is that pre-emptive treatment in primary care would reduce the burden and major costs associated with treatment of overt cryptococcal disease in secondary level care [52], [53]. This would not only reduce overall costs, but would also shift costs to the preventive primary care level.

In conclusion this analysis has shown that CRAG screening would be a highly cost-effective strategy to prevent CM-related mortality among patients initiating ART in South Africa. The most cost-effective, as well as the most practical, approach is provision of high dose fluconazole for all CRAG-positive patients. These findings provide strong justification for the programmatic implementation of CRAG screening in South Africa and are likely to be applicable in other settings of high HIV and CM incidence.

## Supporting Information

Figure S1 **Full Markov Model.** Full Markov model showing the four cryptococcal prevention strategies - 1) no screening or prophylaxis (standard of care or status quo), 2) universal primary prophylaxis with fluconazole 200mg daily, 3) CRAG screening with high-dose fluconazole treatment for all patients testing positive (800mg fluconazole daily for two weeks, followed by 400mg daily for eight weeks, then 200mg daily maintenance) and 4) CRAG screening with lumbar puncture for all patients testing positive, amphotericin-B 1mg/kg/day for two weeks for those with CNS disease and fluconazole 800mg daily for two weeks for those without, in both cases followed by fluconazole 400mg daily for eight weeks, then 200mg daily maintenance as above.
(TIF)
